# Design, Synthesis and SAR Studies of NAD Analogues as Potent Inhibitors towards CD38 NADase

**DOI:** 10.3390/molecules191015754

**Published:** 2014-09-29

**Authors:** Shengjun Wang, Wenjie Zhu, Xuan Wang, Jianguo Li, Kehui Zhang, Liangren Zhang, Yong-Juan Zhao, Hon Cheung Lee, Lihe Zhang

**Affiliations:** 1 State Key Laboratory of Natural and Biomimetic Drugs, School of Pharmaceutical Sciences, Peking University, Beijing 100191, China; E-Mails: wangshengjun@bjmu.edu.cn (S.W.); wangxuan0913@163.com (X.W.); lijianguo.08@163.com (J.L.); kehuizhang@bjmu.edu.cn (K.Z.); zdszlh@bjmu.edu.cn (L.Z.); 2 School of Chemical Biology and Biotechnology, Peking University Shenzhen Graduate School, Shenzhen 518052, China; E-Mails: wilsonzwjdx@126.com (W.Z.); zhaoyongjuan@gmail.com (Y.-J.Z.); leehoncheung@gmail.com (H.C.L.)

**Keywords:** synthesis, NAD analogues, CD38, inhibitors

## Abstract

Nicotinamide adenine dinucleotide (NAD), one of the most important coenzymes in the cells, is a substrate of the signaling enzyme CD38, by which NAD is converted to a second messenger, cyclic ADP-ribose, which releases calcium from intracellular calcium stores. Starting with 2′-deoxy-2′-fluoroarabinosyl-β-nicotinamide adenine dinucleotide (ara-F NAD), a series of NAD analogues were synthesized and their activities to inhibit CD38 NAD glycohydrolase (NADase) were evaluated. The adenosine-modified analogues showed potent inhibitory activities, among which 2′-deoxy-2′-fluoroarabinosyl-β-nicotinamideguanine dinucleotide (ara-F NGD) was the most effective one. The structure-activity relationship of NAD analogues was also discussed.

## 1. Introduction

NAD is an endogenous molecule that is extremely widespread in live cells, it plays very important biological roles as redox coenzyme [1], substrate of ADP-ribosyl transferases [2], ADP-ribosyl cyclases [3], deacetylases [4], DNA ligases [5]; therefore, analogues of NAD may have important biological functions and potential clinical applications. One metabolizing enzyme of NAD in mammals is CD38, a type II or III transmembrane glycoprotein that has important physiological functions. It is firstly identified in lymphocytes by means of monoclonal antibody [6]. Gene knockout studies establish that CD38 plays a critical role in a series of physiological responses such as calcium signaling pathway [7], susceptibility to immune response [8] and social behavior of mice by regulating the plasma level of oxytocin [9]. It is reported that it functions as both an enzyme and a receptor. As a signaling enzyme, it can convert NAD into two messengers, *i.e.*, ADP ribose (ADPR) and cyclic ADP ribose (cADPR). It also catalyzes the synthesis of nicotinic acid adenine dinucleotide phosphate (NAADP) from nicotinamide adenine dinucleotide phosphate (NADP) with a base-exchange reaction in acidic condition. All the three molecules are calcium-mobilizing related [10,11,12]. The catalytic mechanism of multiple reactions catalyzed by CD38 was studied, and both covalent and non-covalent mechanisms have been evidenced [13,14]. It turns out that the same enzyme, CD38, can catalyze the enzymatic reaction through two entirely different reaction pathways depending on the structures of substrates involved [15].

**Figure 1:**
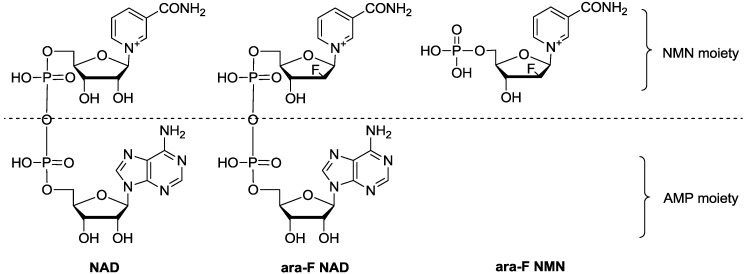
Structures of NAD, ara-F NAD and ara-F NMN.

It is thus of great significance to develop specific and generally applicable inhibitors of CD38. The inhibitors of CD38 can be classified to covalent and non-covalent ones [16,17,18]. The substrate of the enzyme, NAD, consists of two parts, nicotinamide mononucleotide (NMN) and adenosine monophosphate (AMP) (Figure 1). Over the years, 2′-deoxy-2′-fluoroarabinosyl-β-nicotinamide adenine dinucleotide (ara-F NAD) and 2′-deoxy-2′-fluoroarabinosyl-β-nicotinamide mononucleotide (ara-F NMN) were well studied for their inhibitions toward CD38 NADase [19,20], and both of them showed good inhibitory potencies and could be used as probes to investigate the function of CD38 [21].

The structure-activity relationship (SAR) of ara-F NMN analogues has been investigated in our earlier work [16]. To obtain NAD analogues with higher potency and to better understand the molecular mechanism of the binding between CD38 and small molecule inhibitors, various kinds of NAD analogues were synthesized in this study. The analogues were based on the modifications of the three moieties of NAD, *i.e.*, nicotinamide nucleoside, pyrophosphate and adenosine. Their effects on the inhibition of CD38 NADase activities were evaluated. The findings here will be helpful for the development of molecular probes to investigate the function of CD38.

## 2. Results and Discussion

### 2.1. Chemistry

**Scheme 1:**
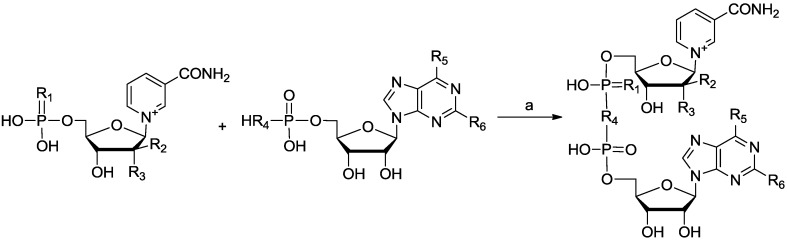
Coupling reaction to synthesize NAD analogues.

The synthesis of NAD analogues could be generalized to three steps, *i.e.*, the synthesis of NMN moiety, AMP moiety and the coupling of the two nucleotides. The formation of the dinucleotide (Scheme 1) was the key step, and it was performed based on previous report [22].

*Reagents and conditions*: (a) CDI, DMF, MeOH, rt, 3 days.

**Table:** 

Entry	R_1_	R_2_	R_3_	R_4_	R_5_	R_6_	Product No.	Yield
1	O	F	H	O	NH_2_	H	**Ara-F NAD**	40%
2	O	CH_3_	F	O	NH_2_	H	**7**	10%
3	S	F	H	O	NH_2_	H	**10**	29%
4	O	F	H	HOPO_3_	NH_2_	H	**11**	6%
5	O	F	H	O	OH	H	**12**	15%
6	O	F	H	O	OH	NH_2_	**13**	10%
7	O	F	H	O	OCH_3_	H	**19**	35%

**Scheme 2:**
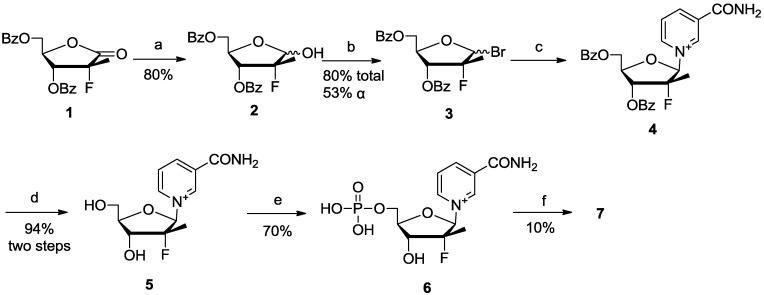
Synthesis of 2′-CH_3_-2′-F NAD (**7**).

*Nicotinamide nucleoside-modified analogues.* The synthesis of the analogues (Scheme 2) started from the commercially available reagent **1**. Reduction of the lactone using lithium tri-*tert*-butoxy- aluminum hydride gave the lactol **2** in about 5:1 β/α anomeric ratio. Conversion of the 1-α-OH isomer to the β form failed, so the next step was performed without separation. The product, 1-bromosugar **3**, was still a mixture of β/α anomers, but it was easily separated to give the pure α-anomer. The conditions that had successfully provided ara-F NMN [23] did not work here for the synthesis of **4**, perhaps due to the methyl substituent that reduced the reactivity of ribofuranose 1′-position. Therefore, the target product **4** was formed by refluxing overnight in acetonitrile. After deprotection of the two hydroxyls using K_2_CO_3_ in methanol, and phosphorylation of the 5′-OH using POCl_3_/TMP, compound **6** was generated. Activation of the phosphate with carbonyldiimidazole (CDI) [22] and coupling with AMP, afforded compound **7**.

*Pyrophosphate-modified analogues*. The ara-F NMN **8** and S-ara-F NMN **9** were obtained following the method reported before [23]. Compound **10** was prepared by coupling **9** with AMP. Compound **8** was activated and coupled with ADP to afford **11** (Scheme 1). Since the triphosphate was more unstable than the diphosphate, **11** was obtained in a poor yield.

*Reagents and conditions*: (a) LiAlH(OBu^t^)_3_, THF; (b) PPh_3_, CBr_4_, DCM; (c) nicotinamide, MeCN, reflux; (d) K_2_CO_3_, MeOH, 3 h; (e) POCl_3_, TMP, 0 °C; (f) CDI, DMF, MeOH, AMP, rt, 3 days.

*Adenosine-modified analogues.* 2′-Deoxy-2′-fluoroarabinosyl-β-nicotinamide hypoxanthine dinucleotide (ara-F NHD, **12**), 2′-deoxy-2′-fluoroarabinosyl-β-nicotinamide guanine dinucleotide (ara-F NGD, **13**) and 2′-deoxy-2′-fluoroarabinosyl-β-nicotinamide 6-O-methylhypoxanthine dinucleotide (6-OMe-ara-F NHD, **19**) were synthesized. Ara-F NHD and ara-F NGD were prepared by coupling ara-F NMN with the commercially available reagents Na_2_·IMP and Na_2_·GMP, respectively (Scheme 1). The products were obtained with relatively low yields, it suggested that the sodium phosphates were not the perfect form of substrates for coupling reaction.

**Scheme 3:**
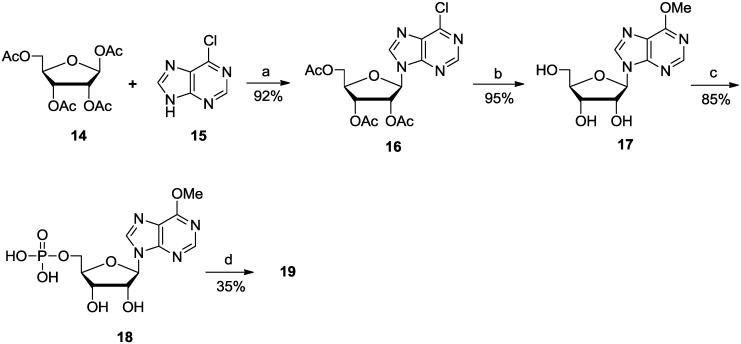
Synthesis of 6-OMe-ara-F NAD (**19**).

*Reagents and conditions*: (a) DBU, TMSOTf, MeCN; (b) 1 M MeONa in MeOH, 0 °C, 1 h; (c) POCl_3_, TMP, 0 °C; (d) CDI, DMF, MeOH, ara-F NMN, rt, 3 days.

6-OMe-ara-F NHD (**19**) was prepared starting from the coupling of tetraacetyl-β-D-ribose (**14**) and 6-chloropurine (**15**) to afford the nucleoside **16** [24], followed by simultaneous remove of the acetyl protecting group and conversion of the 6-chloro to 6-methoxy using 1 M sodium methoxide solution in one step, and then phosphorylation 6-O-Me-AMP (**18**) was performed. The desired compound **19** was generated by treatment of the phosphate-activated ara-F NMN with **18** in DMF (Scheme 3).

**Scheme 4:**
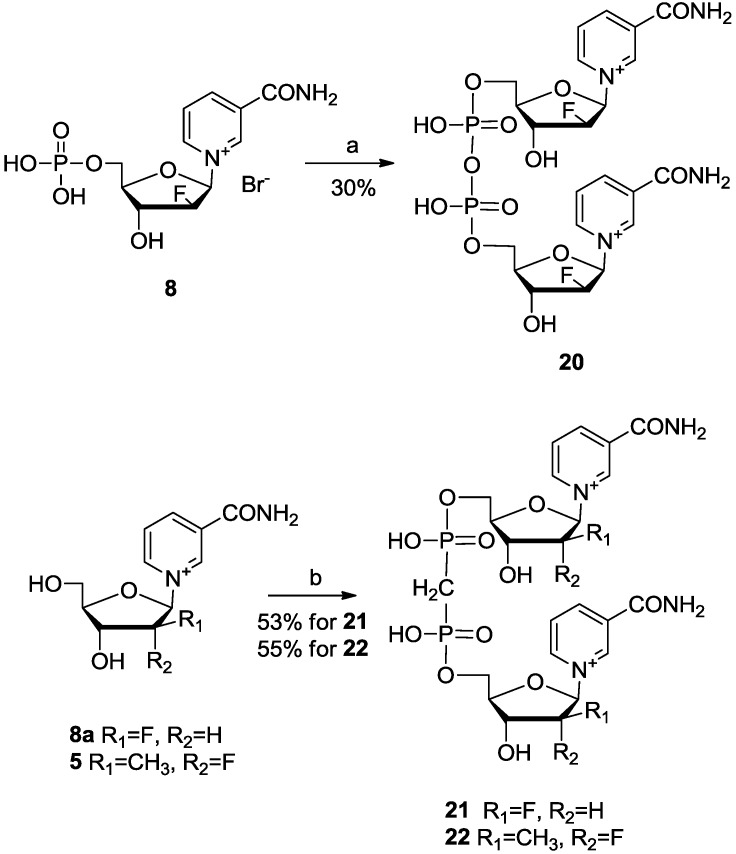
Synthesis of bis(ara-F NMN) (**20**), bis(ara-F NMN)[CH_2_] (**21**) and bis(2′-CH_3_- 2′-F NMN)[CH_2_] (**22**).

We also synthesized three analogues with adenosine replaced by 2′-deoxy-2′-fluoroarabinosyl-β- nicotinamide nucleoside (**8a**) or 2′-deoxy-2′-fluoro-2′-methylarabinosyl-β-nicotinamide nucleoside (**5**). Compound **20** was generated relatively easily by coupling two-equivalents of ara-F NMN **8** together. Compound **21** was obtained by treating compound **8a** [23] with methylenebis (phosphonic dichloride). The same method was used to prepare **22** (Scheme 4).

*Reagents and conditions*: (a) CDI, DMF, MeOH, rt, 3 days; (b) TMP, methylenebis(phosphonic dichloride), 0 °C, 5 h.

### 2.2. Biological Evaluation

*Enzyme inhibition assays*. The inhibitory activities to CD38 NADase of NAD analogues were evaluated by the method reported before [16]. In this method, the inhibitions of NADase activities were calculated from the decrease of the NAD content. Considering the analogues might be detected as NAD because of their structural similarities, the potential interference was evaluated by setting up an independent test using the same method, which showed that the interference was undetectable or negligible at the tested concentrations.

**Figure 2:**
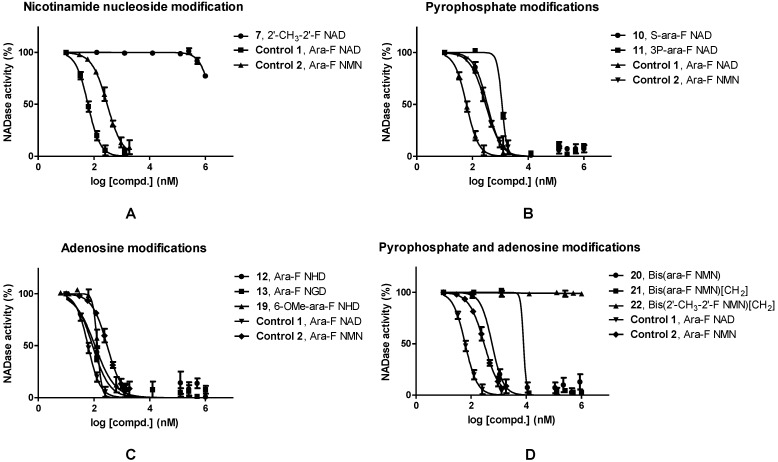
Concentration dependence of inhibitors. Effect of (**A**) nicotinamide nucleoside-modified, (**B**) pyrophosphate-modified, (**C**) adenosine-modified NAD analogues and (**D**) dimers of NMN analogues on CD38 NADase inhibitory activities.

Previous studies on the analogues of ara-F NMN showed that, C-2′ of the furan ring was an important position for the inhibitory activity [16]. Strong inhibitory activity was obtained when C-2′ was substituted by fluoro (ara-F NMN), while double fluoro-substitution resulted in feeble inhibitory activity. To further explore the effect of C-2′ substitution on inhibitory activity, ara-methyl and F were introduced (2′-CH_3_-2′-F NAD, 7). These analogues showed weak inhibition of CD38 NADase (Figure 2A, Table 1). The result implies that C-2′ is very sensitive to substitution. Substituents other than F will make the inhibitory activity decrease or be lost completely.

**Table 1:** Summary of NAD analogues and their inhibitory activities of CD38 NADase. n.s. = no significant inhibitory effect was observed.

Modified Approach	Compd. No.	Compd. Name	IC_50_/nM
**Control 1**	**23**	Ara-F NAD	61.1
**Control 2**	**8**	Ara-F NMN	297
**Nicotinamide nucleoside**	**7**	2′-CH_3_-2′-F NAD	1.81 × 10^6^
**Pyrophosphate**	**10**	S-ara-F NAD	341
	**11**	3P-ara-F NAD	1.15 × 10^3^
**Adenosine**	**12**	Ara-F NHD	109
	**13**	Ara-F NGD	89.3
	**19**	6-OMe-ara-F NHD	133
**Dimer**	**20**	Bis(ara-F NMN)	575
	**21**	Bis(ara-F NMN)[CH_2_]	7.98 × 10^3^
	**22**	Bis(2′-CH_3_-2′-F NMN)[CH_2_]	n.s.

In most cases, the diphosphate linkage of endogenous molecule (cADPR, NAADP, ADPR) is an important moiety for its biological activity, the modification of this part might significantly affect its biological functions, e.g., triphosphate modified cADPR showed increased agonistic activity [25]. We synthesized analogues with phosphorothioate or triphosphate-modified pyrophosphate, *i.e.*, S-ara-F NAD (**10**) and 3P-ara-F NAD (**11**). They showed inhibitory activities with IC_50_ values of 341 nM and 1.15 µM, respectively (Figure 2B, Table 1). The introduction of phosphorothioate or triphosphate will thus result in seriously decreased inhibitory activity.

To study the effects of adenine on the inhibitory activities, we firstly investigated the effects of substituent groups on purine. Three modifications were performed on the adenine ring. All analogues with C-2 or C-6 substituted purines, *i.e.*, ara-F NHD (**12**), ara-F NGD (**13**) and 6-OMe-ara-F NHD (**19**), showed significant inhibitory effects (IC_50_: 89.3 nM to 133 nM; Figure 2C, Table 1). The maintenance of inhibitory activity implies that the adenine moiety tolerates modifications to some extent. Dimers of NMN analogues (**20**–**22**, Figure 2D) could be considered as bis-covalent mimics of ara-F NMN, but these mimics showed obviously decreased inhibitory activities. Bis(ara-F NMN) (**20**) showed an IC_50_ value of 576 nM, which is about one half the potency of its monomer (ara-F NMN). The substitution of pyrophosphate with methylene diphosphonate (compound **21**) led to an even more obvious decrease of inhibitory activity, which implied that the -CH_2_- bridge was not favorable for the interaction of NAD mimics and CD38.

## 3. Experimental Section

### 3.1. General Information

All final products were isolated and purified by high performance liquid chromatography (HPLC), identified by high resolution mass spectrometry (HRMS) and nuclear magnetic resonance (NMR). They were purified at least twice by a C_18_ reversed-phase column (2.2 × 25 cm) equipped on a Gilson HPLC buffer system: H_2_O/MeOH, 1‰ TFA (pH 2–3). HRMS (electrospray ionization) were performed with Bruker APEX IV (Bruker Daltonics Inc., Billerica, MA, USA). ^1^H-NMR and ^13^C-NMR spectra were recorded with a Bruker AVANCE III 400 (Bruker BioSpin AG, Fällanden, Switzerland) instrument at room temperature, CDCl_3_ or D_2_O were used as solvents. Chemical shifts are reported in parts per million downfield from TMS (^1^H and ^13^C). ^31^P-NMR spectra were recorded at room temperature on the Bruker AVANCE III 400. Orthophosphoric acid (85%) was used as external standard. ^19^F-NMR spectra were recorded on the Bruker AVANCE III 400. ^19^F-NMR chemical shifts are reported in ppm with reference to CF_3_COOH (−75.6 relative to -CF_3_) as external standard.

Recombinant CD38, produced with a yeast expression system (Invitrogen, Carlsbad, CA, USA) was used to prepare the recombinant CD38 as reported previously [14]. Briefly, pPICZαA carrying the C-terminus of CD38 (AA46-300, with the four glycosylation sites mutated, N100D, N164D, N209D and N219D) was transformed *Pichia pastoris* yeast, X33. The recombinant CD38 was induced by methanol and purified by phenylsepharose chromatography and cation exchange chromatography (SP column, GE Healthcare, Little Chalfont, UK). All the chemicals used in the enzymatic assays were purchased from Sigma (Santa Clara, CA, USA).

### 3.2. Chemistry

#### General Procedure: Coupling Reaction to Synthesize NAD Analogues

The corresponding lyophilized analogue of NMN (0.l mmol, 1.0 eq.) was dissolved in dried DMF (0.5 mL). Carbonyldiimidazole (CDI, 114 mg, 0.7 mmol, 7.0 eq.) was added under argon atmosphere. The reaction mixture was stirred at room temperature and monitored by HPLC. After 3 h, all the starting material had been consumed and a new peak appeared. A small amount of methanol (50 µL) was added to hydrolyze the excess CDI. The solvent of the reaction mixture was evaporated after 30 min, and then the other nucleoside monophosphate (0.12 mmol, 1.2 eq) which was dissolved in anhydrous DMF (1.5 mL) containing tri-*n*-butylamine (0.12 mmol) was added. The reaction mixture was stirred at room temperature for 3 days under argon atmosphere. The solution was evaporated to dryness. The oily residue was dissolved in water (10 mL) and washed successively with chloroform (3 × 10 mL) and ether (10 mL). The aqueous layer was evaporated again, and dissolved in 1‰ trifluoroacetic acid (TFA) aqueous solution (10 mL), and then purified by HPLC and lyophilized to give dinucleotide as a white cotton-shaped solid.

*3,5-Di-O-benzoyl-2-deoxy-2-fluoro-2-methyl-1-bromide-D-ribofuranose* (**3**). Compound **2** [26] (564 mg, 1.5 mmol, 1.0 eq.) was dissolved in dichloromethane (DCM, 4 mL) under an argon atmosphere. The solution was cooled to −25 °C, and PPh_3_ (555 mg, 2.1 mmol, 1.4 eq.) in DCM (3 mL) were added, stirred for 15 min, then CBr_4_ (750 mg, 2.29 mmol, 1.5 eq.) in DCM (2 mL) was added. After reacting for 0.5 h at −17 °C, silica gel (900 mg) was added to the mixture, which was filtered and washed with DCM. The combined filtrates were concentrated under reduced pressure and the residue were purified by column chromatography (petroleum ether-ethyl acetate = 150:1) to give **3** as a colorless oil (α, 350 mg, 53%). ^1^H-NMR (400 MHz, CDCl_3_) δ 8.21–7.98 (m, 4H), 7.68–7.38 (m, 6H), 6.34 (s, 1H), 5.30–5.27 (m, 1H), 4.87 (m, 1H), 4.77 (dd, *J* = 12.5, 3.2 Hz, 1H), 4.63 (dd, *J* = 12.5, 4.5 Hz, 1H), 1.72 (d, *J* = 21.5 Hz, 3H).

*2′-Deoxy-2′-fluoro-2′-methyl-β-nicotinamide ribofuranoside* (**5**)*.* Compound **3** (330 mg, 0.76 mmol, 1.0 eq.) was dissloved in anhydrous acetonitrile (MeCN, 3 mL), nicotinamide (463 mg, 0.38 mmol, 5.0 eq.) was added and the mixture was refluxed overnight. The solvent of the reaction mixture was evaporated to give a yellow oil. The mixture was dissolved in MeOH (4 mL), K_2_CO_3_ (126 mg, 0.91 mmol, 1.2 eq.) was added and the mixture stirred for 2 h at room temperature. The mixture was concentrated under reduced pressure and the residue were purified by column chromatography (DCM-MeOH = 3:1), to give compound **5** (250 mg, 94%) as a pale yellow vesicular solid. ^1^H-NMR (400 MHz, D_2_O) δ 9.32 (s, 1H), 9.11 (d, *J* = 6.3 Hz, 1H), 8.99 (d, *J* = 8.2 Hz, 1H), 8.26 (t, *J* = 7.2 Hz, 1H), 6.52 (d, *J* = 17.1 Hz, 1H), 4.63–4.56 (m, 1H), 4.30 (m, 1H), 4.05–3.97 (m, 1H), 3.78 (dd, *J* = 13.1, 4.3 Hz, 1H), 1.58 (d, *J* = 22.8 Hz, 3H); ^19^F-NMR (376 MHz, D_2_O) δ −172.73.

*2′-Deoxy-2′-fluoro-2′-methyl-β-nicotinamide mononucleotide* (**6**). Compound **5** (176 mg, 0.50 mmol, 1.0 eq.) was dissolved in trimethyl phosphate (TMP, 2.5 mL), and POCl_3_ (0.23 mL, 2.50 mmol, 5.0 eq.) was added slowly to the reaction mixture under ice bath cooling. The mixture was stirred for 2 h at 0 °C, aqueous sodium hydroxide was then added to neutralize excess acid to a final pH of 7. The solution was reduced to dryness, and then the gummy residue was dissolved in water (10 mL) and extracted with ethyl acetate (3 × 10 mL). The aqueous layer was evaporated again, and dissolved in 10 mL of 1‰ aqueous TFA solution. After purification by HPLC and lyophilization, **6** (160 mg, 69%) was generated as a white cotton-shaped solid. ^1^H-NMR (400 MHz, D_2_O) δ 9.32 (s, 1H), 9.10 (d, *J* = 6.1 Hz, 1H), 8.98 (d, *J* = 8.1 Hz, 1H), 8.24 (dd, *J* = 7.7, 6.7 Hz, 1H), 6.53 (d, *J* = 17.2 Hz, 1H), 4.70–4.66 (m, 1H), 4.39 (dd, *J* = 24.0, 9.3 Hz, 1H), 4.32 (ddd, *J* = 12.2, 5.9, 2.0 Hz, 1H), 4.16–4.08 (m, 1H), 1.56 (d, *J* = 22.8 Hz, 3H); ^31^P-NMR (162 MHz, D_2_O) δ 0.02; ^19^F-NMR (376 MHz, D_2_O) δ −75.63, −173.38.

*2′-Deoxy-2′-fluoro-2′-methyl-β-nicotinamide adenine dinucleotide* (2′-CH_3_-2′-F NAD, **7**). Compound **6** (TFA salt, 0.10 mmol, 46 mg, 1.0 eq.) and AMP (0.12 mmol, 42 mg, 1.2 eq.) were reacted following the general procedure to yield **7** (TFA salt) (8 mg, 10%) as a white cotton-shaped solid. ^1^H-NMR (400 MHz, D_2_O) δ 9.30 (s, 1H), 9.13 (d, *J* = 5.9 Hz, 1H), 8.96 (d, *J* = 8.0 Hz, 1H), 8.55 (s, 1H), 8.37 (s, 1H), 8.24 (t, *J* = 7.1 Hz, 1H), 6.57 (d, *J* = 17.3 Hz, 1H), 6.02 (d, *J* = 5.4 Hz, 1H), 4.73–4.63 (m, 2H), 4.53–4.37 (m, 3H), 4.33 (s, 1H), 4.21 (m, 3H), 1.56 (d, *J* = 22.8 Hz, 3H); ^31^P-NMR (162 MHz, D_2_O) δ −11.33; ^19^F-NMR (376 MHz, D_2_O) δ −75.71, −173.82; HRMS (ESI-TOF^+^) calcd for C_22_H_29_FN_7_O_13_P_2_ [(M+H)], 680.1277; found, 680.1281.

*2′-Deoxy-2′-fluoro-5′-thio-phosphate-arabinofuranoside-β-nicotinamide adenine dinucleotide* (S-ara-F NAD, **10**). Compound **9** (TFA salt) [16] (0.10 mmol, 47 mg, 1.0 eq.) and AMP (0.12 mmol, 42 mg, 1.2 eq.) were reacted following the general procedure to yield **10** (TFA salt) (23 mg, 29%) as a white cotton-shaped solid. ^1^H-NMR (400 MHz, D_2_O) δ 9.30 (m, 2H), 8.87 (d, *J* = 7.9 Hz, 1H), 8.53 (s, 1H), 8.34 (s, 1H), 8.27–8.20 (m, 1H), 6.64 (dd, *J* = 8.5, 4.8 Hz, 1H), 6.04 (d, *J* = 5.3 Hz, 1H), 5.48 (dt, *J* = 51.3, 4.7 Hz, 1H), 4.69 (t, *J* = 5.3 Hz, 1H), 4.57 (dt, *J* = 17.6, 5.2 Hz, 1H), 4.52–4.43 (m, 1H), 4.41–4.33 (m, 2H), 4.32–4.17 (m, 4H); ^31^P-NMR (162 MHz, D_2_O) δ 44.14 (d, *J* = 27.0 Hz), −12.32 (d, *J* = 27.4 Hz); ^19^F-NMR (376 MHz, D_2_O) δ −75.79, −199.16; HRMS (ESI-TOF^+^) calcd for C_21_H_27_FN_7_O_12_P_2_S [(M+H)], 682.0892; found, 682.0881.

*P^1^-(Adenosine)-P^3^-(2′-deoxy-2′-fluoro-β-nicotinamide arabinofuranoside) triphosphate* (3P-ara-F NAD, **11**). Ara-F NMN (TFA salt) [23] (0.10 mmol, 45 mg, 1.0 eq.) and ADP (0.12 mmol, 51 mg, 1.2 eq.) were reacted following the general procedure to yield **11** (TFA salt) (5.2 mg, 6.0%) as a white cotton-shaped solid. ^1^H-NMR (400 MHz, D_2_O) δ 9.38 (s, 1H), 9.28 (D, *J* = 5.4 Hz, 1H), 8.92 (d, *J* = 7.6 Hz, 1H), 8.55 (s, 1H), 8.36 (s, 1H), 8.27 (t, *J* = 6.4 Hz, 1H), 6.67 (s, 1H), 6.07 (d, *J* = 5.3 Hz, 1H), 5.50 (d, *J* = 51.2 Hz, 1H), 4.66 (d, *J* = 5.0 Hz, 2H), 4.47 (s, 1H), 4.38 (s, 2H), 4.28 (m, *J* = 39.1 Hz, 4H) ^19^F-NMR (376 MHz, D_2_O) δ −75.75, −199.40; HRMS (ESI-TOF^+^) calcd for C_21_H_28_FN_7_O_16_P_3_ [(M+H)], 746.0784; found, 746.0788.

*2′-Deoxy-2′-fluoro-arabinosyl-β-nicotinamide hypoxanthine dinucleotide* (ara-F NHD, **12**). Ara-F NMN (TFA salt) (0.10 mmol, 45 mg, 1.0 eq.) and Na_2_·IMP (0.12 mmol, 47 mg, 1.2 eq.) were reacted following the general procedure to yield **12** (TFA salt) (7.8 mg, 15%) as a white cotton-shaped solid. ^1^H-NMR (400 MHz, D_2_O) δ 9.35 (s, 1H), 9.24–9.15 (m, 2H), 8.90 (d, *J* = 8.0 Hz, 1H), 8.28–8.18 (m, 2H), 6.64 (dd, *J* = 10.3, 4.5 Hz, 1H), 6.13 (d, *J* = 3.5 Hz, 1H), 5.45 (dt, *J* = 51.2, 4.5 Hz, 1H), 4.63–4.51 (m, 2H), 4.41–4.13 (m, 7H); ^31^P-NMR (162 MHz, D_2_O) δ −11.47; ^19^F-NMR (376 MHz, D_2_O) δ −75.78, −199.16; HRMS (ESI-TOF^+^) calcd for C_21_H_26_FN_6_O_14_P_2_ [(M+H)], 667.0961; found, 667.0978.

*2′-Deoxy-2′-fluoro-arabinosyl-β-nicotinamide guanine dinucleotide* (ara-F NGD, **13**). Ara-F NMN (TFA salt) (0.10 mmol, 45 mg, 1.0 eq.) and Na_2_·GMP (0.12 mmol, 49 mg, 1.2 eq.) were reacted following the general procedure to yield **13** (TFA salt) (8.0 mg, 10%) as a white cotton-shaped solid. ^1^H-NMR (400 MHz, D_2_O) δ 9.43 (s, 1H), 9.30 (d, *J* = 6.2 Hz, 1H), 9.07–8.95 (m, 2H), 8.35–8.28 (dd, *J* = 7.6, 6.8 Hz, 1H), 6.72 (dd, *J* = 9.8, 4.7 Hz, 1H), 6.01 (d, *J* = 3.8 Hz, 1H), 5.55 (dt, *J* = 51.4, 4.7Hz, 1H), 4.70–4.58 (m, 2H), 4.50–4.18 (m, 7H); ^31^P-NMR (162 MHz, D_2_O) δ −11.45; ^19^F-NMR (376 MHz, D_2_O) δ −75.74, −199.21; HRMS (ESI-TOF^+^) calcd for C_21_H_27_FN_7_O_14_P_2_ [(M+H)], 682.1070; found, 682.1074.

*2′-Deoxy-2′-fluoro-arabinosyl-β-nicotinamide 6-O-methyl-hypoxanthine dinucleotide* (6-OMe-ara-F NHD, **19**). Ara-F NMN (TFA salt, 0.10 mmol, 45 mg, 1.0 eq.) and 6-OMe-IMP [27] (0.12 mmol, 44 mg, 1.2 eq.) were reacted following the general procedure to yield **19** (TFA salt) (28 mg, 35%) as a white cotton-shaped solid. ^1^H-NMR (400 MHz, D_2_O) δ 9.30 (s, 1H), 9.21–9.10 (m, 2H), 8.84 (d, *J* = 8.0 Hz, 1H), 8.55 (s, 1H), 8.18 (t, *J* = 6.8 Hz, 1H), 6.59 (dd, *J* = 9.5, 4.1 Hz, 1H), 6.16 (d, *J* = 2.8 Hz, 1H), 5.53–5.35 (m, 1H), 4.62 (t, *J* = 3.8 Hz, 1H), 4.52 (dt, *J* = 9.7, 4.6 Hz, 1H), 4.38 (t, *J* = 4.5 Hz, 1H), 4.36–4.14 (m, 6H), 4.12 (s, 3H); ^31^P-NMR (162 MHz, D_2_O) δ −11.40; ^19^F-NMR (376 MHz, D_2_O) δ −75.72, −199.11; HRMS (ESI-TOF^+^) calcd for C_22_H_28_FN_6_O_14_P_2_ [(M+H)], 681.1117; found, 681.1118.

*Bis(2′-deoxy-2′-fluoro-β-nicotinamide-arabinosyl) pyrophophate* (Bis(ara-F NMN), **20**). Ara-F NMN (TFA salt, 0.10 mmol, 45 mg, 1.0 eq.) and another ara-F NMN (TFA salt, 0.12 mmol, 54 mg, 1.2 eq.) were added respectively following the general procedure to yield **20** (TFA salt) (27 mg, 30%) as a white cotton-shaped solid. ^1^H-NMR (400 MHz, D_2_O) δ 9.41 (s, 2H), 9.28 (d, *J* = 6.2 Hz, 2H), 8.97 (d, *J* = 8.1 Hz, 2H), 8.29 (dd, *J* = 7.9, 6.5 Hz, 2H), 6.71 (dd, *J* = 9.7, 4.7 Hz, 2H), 5.52 (dt, *J* = 51.3, 4.7 Hz, 2H), 4.61 (dt, *J* = 17.7, 5.0 Hz, 2H), 4.41 (m, 4H), 4.34–4.27 (m, 2H); ^31^P-NMR (162 MHz, D_2_O) δ −11.45; ^19^F-NMR (376 MHz, D_2_O) δ −75.68, −199.09; HRMS (ESI-TOF^+^) calcd for C_22_H_28_F_2_N_4_O_13_P_2_ [(M+H)], 655.1012; found, 655.1007.

*Bis(2′-deoxy-2′-fluoro-β-nicotinamide-arabinosyl)-methylenediphosphonate* (Bis(ara-F NMN)[CH_2_], **21**). Compound **8a** (70 mg, 0.21 mmol, 2.0 eq.) was dissolved in TMP (2.5 mL), (Cl_2_PO)_2_CH_2_ (26 mg, 0.11 mmol, 1.0 eq.) was added to the reaction mixture under ice bath cooling. The mixture was stirred for 5 h at 0 °C, then aqueous sodium hydroxide was added to the neutralize excess acid to pH = 7. The solution was reduced to dryness, and then the gummy residue was dissolved in water (10 mL) and extracted with ethyl acetate (3 × 10 mL). The aqueous layer was evaporated again, and dissolved in 1‰ TFA aqueous solution (10 mL). After purification by HPLC and lyophilization, **21** (TFA salt) (50 mg, 53%) was generated as a white cotton-shaped solid. ^1^H-NMR (400 MHz, D_2_O) δ 9.43 (s, 2H), 9.25 (d, *J* = 6.1 Hz, 2H), 8.96 (d, *J* = 8.1 Hz, 2H), 8.26 (dd, *J* = 7.6, 6.8 Hz, 2H), 6.71 (dd, *J* = 10.2, 4.5 Hz, 2H), 5.51 (dt, *J* = 51.3, 4.5 Hz, 2H), 4.59 (dt, *J* = 17.5, 4.8 Hz, 2H), 4.41 (dd, *J* = 14.4, 9.5 Hz, 4H), 4.34–4.26 (m, 2H), 2.50 (t, *J* = 20.4 Hz, 2H); ^31^P-NMR (162 MHz, D_2_O) δ 18.35; ^19^F-NMR (376 MHz, D_2_O) δ −75.69, −198.97; HRMS (ESI-TOF^+^) calcd for C_23_H_30_F_2_N_4_O_12_P_2_ [(M+H)], 653.1220; found, 653.1224.

*Bis(2′-deoxy-2′-fluoro-2′-methyl-β-nicotinamide)-methylenediphosphonate* (Bis(2′-CH_3_-2′-F NMN)[CH_2_], **22**). Compound **22** was synthesized from **5** using the same procedure as for **21**. Yield 55% as a white cotton-shaped solid. ^1^H-NMR (400 MHz, D_2_O) δ 9.35 (s, 2H), 9.13 (d, *J* = 5.7 Hz, 2H), 8.99 (d, *J* = 8.1 Hz, 2H), 8.26 (dd, *J* = 7.9, 6.5 Hz, 2H), 6.58 (d, *J* = 17.3 Hz, 2H), 4.72 (d, *J* = 9.0 Hz, 2H), 4.52–4.38 (m, 4H), 4.25–4.17 (m, 2H), 2.42 (t, *J* = 20.4 Hz, 2H), 1.57 (d, *J* = 22.8 Hz, 6H); ^31^P-NMR (162 MHz, D_2_O) δ 18.59; ^19^F-NMR (376 MHz, D_2_O) δ −75.70, −173.53; HRMS (ESI-TOF^+^) calcd for C_25_H_34_F_2_N_4_O_12_P_2_ [(M+H)], 681.1533; found, 681.1517.

### 3.3. Enzyme Activity Assay

To study the inhibition of the NAD-glycohydrolase (NADase) activity of CD38 by the compounds, an enzymatic assay was performed [16]. Briefly, different concentrations of the compound were mixed with recombinant CD38 (0.3 ng) and bovine serum albumin (1 µg) in a 16 µL-reaction mixture, then incubated in the dark for 2 hours at room temperature. NAD was added to the mixture to a final concentration of 2.5 µM to start the reaction. Aliquots (4 µL) of the reaction mixtures were withdrawn at 0, 4, 8 and 12 min, and the reaction was stopped by adding 4 µL of 0.6 M HCl, followed by neutralization with 8 µL of 0.5 M phosphate buffer, pH 8. The subsequent cycling reaction was conducted in 96-well plate with 16 µL of the sample mixed with 100 µL of the reagent containing 2% ethanol, 100 µg/mL alcohol dehydrogenase, 20 µM resazurin, 11 µg/mL diaphorase, 10 µM flavin mononucleotide and 100 mM sodium phosphate, pH 7. The cycling reaction was allowed to proceed for 10 min and the rate of fluorescence increase of resorufin (with excitation at 544 nm and emission at 590 nm) was measured using the Infinite M200 fluorescence plate reader (TECAN, Grödig, Austria). The relative NAD content was measured from the slope of fluorescence increase and the NAD-glycohydrolase activity was calculated from the decrease of NAD content at 0, 4, 8 and 12 min after the reaction started. As a control, each compound was directly added to the cycling reaction system with corresponding concentration to evaluate the potential interference to the cycling assay itself. IC_50_ values of inhibitors were calculated by the GraphPad Prism 5 software (GraphPad Software, Inc., La Jolla, CA, USA).

## 4. Conclusions

**Figure 3:**
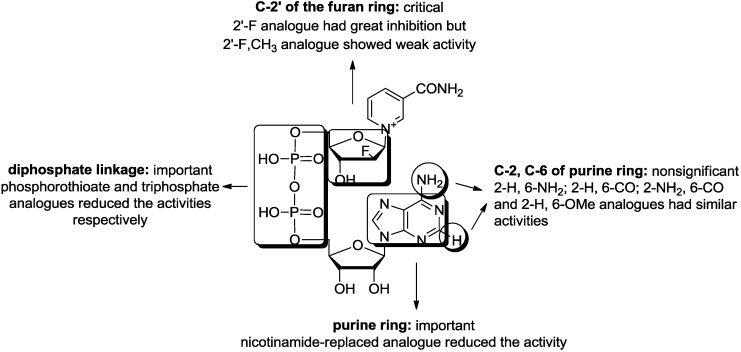
Preliminary structure-activity relationship for NAD analogues.

NAD analogues with ribose, nucleobase, or pyrophosphate modifications were designed, prepared and their inhibitory activities against CD38 NADase were evaluated. SAR studies (Figure 3) revealed that three analogues (compounds **12**, **13** and **19**) with a modified purine ring showed higher activities than the others, while the analogues with the purine ring replaced by nicotinamide showed obviously decreased activities, suggesting that the purine ring is important for the activity but subtle substituent changes at C-2 and C-6 of the purine ring did not significantly affect the activity. The charge, length and flexibility of the pyrophosphate bridge are important for retaining inhibitory activity; the replacement by phosphorothioate or triphosphate reduces the activity. A single fluoro substituent at C-2′ of the furan ring is critical for activity, and 2′-disubstitution, such as the introduction of fluoro and methyl leads to weak activity. The findings of this study present some insights into the structure-activity relationship of NAD analogues, which should be helpful for the discovery of more active probes to investigate NAD-related biological effects.
